# Tomato brown rugose fruit virus in aqueous environments – survival and significance of water-mediated transmission

**DOI:** 10.3389/fpls.2023.1187920

**Published:** 2023-06-02

**Authors:** Nataša Mehle, Katarina Bačnik, Irena Bajde, Jakob Brodarič, Adrian Fox, Ion Gutiérrez-Aguirre, Miha Kitek, Denis Kutnjak, Yue Lin Loh, Olivera Maksimović Carvalho Ferreira, Maja Ravnikar, Elise Vogel, Christine Vos, Ana Vučurović

**Affiliations:** ^1^ Department of Biotechnology and Systems Biology, National Institute of Biology, Ljubljana, Slovenia; ^2^ School for Viticulture and Enology, University of Nova Gorica, Vipava, Slovenia; ^3^ Jožef Stefan International Postgraduate School, Ljubljana, Slovenia; ^4^ Fera Science Ltd., York, United Kingdom; ^5^ School of Natural and Environmental Sciences, Newcastle University, Newcastle upon Tyne, United Kingdom; ^6^ Biotechnical Faculty, University of Ljubljana, Ljubljana, Slovenia; ^7^ Scientia Terrae Research Institute VZW, Sint-Katelijne-Waver, Belgium; ^8^ De Ceuster Meststoffen NV (DCM), Grobbendonk, Belgium

**Keywords:** tomato brown rugose fruit virus, water-linked epidemiology, survival, tomato, hydroponics

## Abstract

Tomato brown rugose fruit virus (ToBRFV) has recently emerged as a major disease of tomatoes and peppers. ToBRFV is a seed- and contact-transmitted virus. In Slovenia, ToBRFV RNA was detected in samples of wastewater, river, and water used to irrigate plants. Even though the source of detected RNA could not be clearly established, this raised the question of the significance of the detection of ToBRFV in water samples and experimental studies were performed to address this question. The data presented here confirm that the release of virus particles from the roots of infected plants is a source of infectious ToBRFV particles in water and that the virus can remain infective up to four weeks in water stored at room temperature, while its RNA can be detected for much longer. These data also indicate that irrigation with ToBRFV-contaminated water can lead to plant infection. In addition, it has been shown that ToBRFV circulated in drain water in commercial tomato greenhouses from other European countries and that an outbreak of ToBRFV can be detected by regular monitoring of drain water. A simple method for concentrating ToBRFV from water samples and a comparison of the sensitivity of different methods, including the determination of the highest ToBRFV dilution still capable of infecting test plants, were also investigated. The results of our studies fill the knowledge gaps in the epidemiology and diagnosis of ToBRFV, by studying the role of water-mediated transmission, and provide a reliable risk assessment to identify critical points for monitoring and control.

## 1 Introduction

Tomato brown rugose fruit virus (ToBRFV, genus *Tobamovirus*, family *Virgaviridae*) was first detected in field-grown tomatoes, which showed typical mosaic symptoms as well as leaf narrowing and yellow or brown rugose spots on the fruits (Salem et al., 2016; Luria et al., 2017). This resulted in huge yield losses in Israel in 2014 (Luria et al., 2017), and in Jordan in 2015 (Salem et al., 2016). After its initial discovery in Israel and Jordan, it was reported in more than 50 countries (EPPO GD, 2022). Therefore, ToBRFV is considered a pathogen that is changing global tomato production (Caruso et al., 2022), which takes place on more than five billion hectares and tomato is considered one of the most important vegetables (FAOSTAT, 2020). Economic losses due to ToBRFV infection have also been reported for pepper plants (OEPP/EPPO, 2020). The disease incidence in affected crops was estimated from 50 to 100% (Salem et al., 2016; Alkowni et al., 2019), while observed yield reduction was 10-55% (Avni et al., 2019). Infected plants exhibit mild to severe mosaic, and deformation of leaves, while the fruits may develop brown rugose (rough) patches, marbling, and growth deformation. The rapid spread of ToBRFV to different countries across multiple continents in less than a decade after its emergence was most likely due to the transfer of infested seeds from its place of origin (Caruso et al., 2022). Seed transmission rates are low, however, infection can have a major impact on intensive greenhouse production (Oladokun et al., 2019; Salem et al., 2022). Once established in the production facility, the virus can spread rapidly through plant-to-plant contact (Panno et al., 2020) and, during the course of common cultivation practices, through wounds on leaves or on the roots of seedlings (e.g., after transplanting) (Salem et al., 2016). Like other tobamoviruses, ToBRFV has extremely stable virions (Zhang et al., 2022). Due to their virion stability, tobamoviruses exhibit high persistence in soil, as well as irrigation and drainage water, and remain infectious over long periods of time (Li et al., 2016; Caruso et al., 2022). In previous studies, tobamoviruses have been detected in different environmental samples, including soil (Fillhart et al., 1998), clouds (Castello et al., 1995), and water (Kuroda et al., 2015). Additionally, sequences of tobamoviruses have been detected in different environmental waters, including drinking water (Haramoto et al., 2013), ballast water (Kim et al., 2015), irrigation systems (Boben et al., 2007), and raw and urban sewage (Cantalupo et al., 2011; Fernandez-Cassi et al., 2018). A high diversity of tobamoviral species was reported for reclaimed water (Rosario et al., 2009) and wastewater influents and effluents from a central Slovenian wastewater treatment plant, where some tobamoviruses were confirmed to be infective (Bačnik et al., 2020). Sequences of ToBRFV were detected in wastewater influent sample, although the virus at that time had not yet been reported as infecting plants in Slovenia (Bačnik et al., 2020). This raises questions about its origin, the possibility of its unnoticed occurrence, and the risks of its transmission through water, as well as about the possible use of such water sources in agriculture for irrigation during water shortages.

Modern agriculture requires the use of irrigation in crop production. According to reports, water use in agricultural production accounts for >80% of global water consumption (Xinchun et al., 2017). Moreover, global food security may be threatened by severe water scarcity due to climate change (Murtaza et al., 2019). Agricultural producers use freshwater, wastewater, groundwater, and surface water for irrigation. The use of wastewater in agriculture is considered one of the ways to overcome water scarcity; however, water from alternative sources such as wastewater is sometimes of low quality and requires continuous management and monitoring (Murtaza et al., 2019).

Water-mediated transmission may accelerate global disease emergence and ecosystem impacts for a wide range of crops (Mehle et al., 2018), including tomato and pepper. This is especially true, when hydroponic systems are widely used for the production of some crop species because they require significantly less water, and can contribute to solving the global problem of water scarcity (Sambo et al., 2019). Crop production in soilless cultures using open or closed hydroponic systems has been increasing worldwide. Soilless culture is an alternative for plant growers facing soil-related problems such as nematodes and pathogens, as well as nutrient imbalances (Stanghellini and Rasmussen, 1994; Schnitzler, 2004). In contrast, the use of circulating nutrient solutions in hydroponic systems has the potential for the rapid and effective spread of water-transmissible plant pathogens throughout the crop (whether the pathogens are contained in the original water source or enter the water *via* the distribution pathway), increasing the likelihood of disease outbreaks if the system is not intensively managed (Stewart-Wade, 2011). Therefore, the risk of spreading and the required management of plant viruses, which often lead to crop losses, needs to be assessed before recirculating used water (Bandte et al., 2009).

Previous studies have shown that various tobamoviruses can be transmitted from contaminated soil to the upper parts of plants through the roots (Broadbent, 1965; Fletcher, 1969; Allen, 1981; Koenig, 1986; Pares et al., 1992; Antignus et al., 2005; Li et al., 2016; Avni et al., 2019). In addition, tomato mosaic virus has been shown to persist in root debris in the soil for almost two years and to be infectious for even slightly longer in dry soils (Fletcher, 1969; Broadbent, 1976).

To date, there have been few comprehensive studies on water-mediated transmission of plant viruses, and none have been conducted on emerging tobamoviruses. In a previous study, it has been shown that pepino mosaic virus (PepMV, genus *Potexvirus*), potato virus Y (PVY, genus *Potyvirus*), and potato spindle tuber viroid (PSTVd, genus *Pospiviroid*) can be released from plant roots into the nutrient solution, where they remain infectious, and are able to infect healthy plants through the roots and eventually spread to the green parts, where they can be detected after several months (Mehle et al., 2014). It should be noted, however that the virus/viroid was not detected in the green parts of all plants examined in the study by Mehle et al. (2014), suggesting that water may not be the main route of transmission of PepMV, PVY, and PSTVd between plants. However, it may allow the infection of individual plants, whereupon both viruses and viroids can spread rapidly and effectively to neighboring plants, either mechanically, by vectors or by other means. As is the case for PepMV, PVY, PSTVd, tobamoviruses are also very stable and easily transmissible, which must be taken into account when investigating the potential of water as a transmission route. Furthermore, when interpreting the results, it should be noted that the infection of plants with stable and easily transmissible viruses can occur not only through the roots, but also through the upper parts of the plant when contaminated water is used for irrigation (e.g., through small wounds that may appear on the leaf surfaces during irrigation, by wind action, or by the application of common agricultural practices) (Mehle and Ravnikar, 2012).

The main objective of this study was to explore the water-linked epidemiology of ToBRFV. Experiments were designed to reveal the possible role of water contaminated with ToBRFV in hydroponic systems as well as in conventional production systems. The main concern was to answer the following questions: (i) can ToBRFV be released from the roots of infected plants into irrigation water, (ii) how long does ToBRFV remain infectious in water, and (iii) can ToBRFV infect plants through the roots when plants are irrigated with contaminated water? In addition, the utility of water monitoring for ToBRFV in commercial tomato greenhouses was evaluated, and some improvements were made to the diagnostic procedure for water analysis.

## 2 Materials and methods

### 2.1 Detection of ToBRFV in different environmental water samples

Fourteen samples (5 liters) of wastewater, river water, and irrigation water from different locations in Slovenia from 2017 to 2022 (**Supplementary Table 1**) were filtered through filter paper and cellulose acetate membranes with a pore size of 0.8 µm (Sartorius, Germany) to remove larger particles. Concentration of each sample was performed using an 8-ml convective interaction media quaternary amine (CIM QA) monolith column (BIA Separations, Slovenia) on an AKTA Purifier 100 FPLC system (GE Healthcare, USA) as previously described (Bačnik et al., 2020). RNA was extracted from the concentrated and non-concentrated water samples using QIAamp Viral RNA mini kit (Qiagen, USA), stored at -20°C, and then tested for the presence of ToBRFV by one step real-time quantitative reverse transcription PCR (RT-qPCR) using primers and probes from Menzel and Winter (2021) and ISHI-Veg (2019) as described in EPPO standard PM 7/146(1) (EPPO, 2021) (hereafter: M&W RT-qPCR and ISF-ISHI-Veg RT-qPCR, respectively). As a control of the RNA extraction procedure and to account for potential inhibition of the qPCR reaction, luciferase control RNA (Promega) was added to each sample and to a negative buffer control (2 ng per sample) immediately prior to the RNA extraction and then tested with RT-qPCR using luciferase RNA-specific primers and probe (Toplak et al., 2004). Analysis of luciferase control RNA showed that the extractions were successful and the inhibition was not present. Negative controls were included in all concentration steps, RNA extractions, and RT-qPCR runs to monitor possible contamination during the procedures. Analysis of these negative controls did not reveal any contamination during the process. In addition, for confirmation, RNA from the 2021 concentrated water sample was further analyzed by conventional RT-PCR with ToBRFV-specific primers (Panno et al., 2019) and by nested RT-PCR with generic tobamovirus primers (Dovas et al., 2004), and the obtained amplicons were analyzed by Sanger sequencing.

### 2.2 Handling of test plants and water samples from greenhouse experiments

Experiments were performed in a quarantine greenhouse with temperatures of 22 ± 2°C during the light period (16 h) and 19 ± 2 °C during the dark period (8 h). ToBRFV isolate (DSMZ PV-1236) was propagated on tomato (*Solanum lycopersicum*) cv. Moneymaker. Briefly, two to three fully developed lower leaves of tomato were dusted with Carborundum powder (400 mesh, VWR Chemicals, the Netherlands) and then inoculated with the extract of leaf inoculum prepared in a 0.02 M phosphate buffer (pH 7.4) containing 2% polyvinylpyrrolidone 10,000. Mechanical inoculation of test plants with water or nutrient solution samples was performed in such a way that a few drops (approximately 300-500 μl) of these samples were used as inoculum. Five to ten minutes after inoculation, the test plants were rinsed with tap water to remove the abrasive and kept in a quarantine greenhouse. Symptom development on test plants was monitored weekly, and infection was confirmed on newly developed leaves using a rapid one-step assay based on lateral flow immunochromatography (ImmunoStrip^®^, Agdia, USA) (hereafter: LFD) according to the manufacturer’s instructions or by testing extracted RNA using M&W RT-qPCR.

Total RNA was extracted from leaf material (approximately 200 mg) using RNeasy Plant mini kit (Qiagen, USA), following the manufacturer recommendations, with minor modifications: specifically, without using 2-mercaptoethanol, and performing the final RNA elution with two consecutive washes with 50 µl (total of 100 µl) of RNase-free water pre-warmed to 65°C. To assess the quality of the RNA in the extractions, RNA samples were tested with RT-qPCR using *nad5*-specific primers and a probe (Menzel et al., 2002), and only RNA samples with a Cq value for *nad5* of less than 33 were analyzed further. RNA from water and nutrient solution samples was extracted using QIAamp Viral RNA mini kit, and its quality was measured as described above.

Periodic testing with M&W RT-qPCR of control plants grown in the same chamber of the quarantine greenhouse as the experimental plants were used to check the adventitious spread of ToBRFV during greenhouse manipulations. Due to limited space in the quarantine greenhouses, the tops of tomato plants were occasionally pruned to keep them at a maximum height up to one and half meter.

### 2.3 Comparison of different approaches for the detection of ToBRFV RNA in water

Three trays of three tomato plants each were placed in a greenhouse chamber: one with all three plants inoculated with ToBRFV, one with just one plant out of three inoculated with ToBRFV, and one with three healthy tomato plants. Plants were grown in Grodan rockwool cubes (100 mm cubes, Grodan, Netherlands) floating on nutrient solution (Johnson et al., 1994). The nutrient solution was sampled three times per week (each time 3 × 50 ml per tray) while the level of nutrient solution was kept constant. Nutrient solution samples were stored at 4°C for six months, and then M&W RT-qPCR was performed for each nutrient solution sample. The nutrient solution was analyzed using RT-qPCR in three different ways: (i) directly from non-concentrated water sample without RNA extraction; (ii) RNA extracted with QIAamp Viral RNA mini kit from non-concentrated sample; (iii) RNA extracted from the concentrated sample from water sample concentrated using Centricon Plus-70 Centrifugal Filter Units, 10 kDa (Millipore, Germany, UFC701008). For concentration, samples were first centrifuged at 3,200 g for 10 min, and the supernatant (2 × 40 ml) was applied to Centricon units. The Centricon units were then centrifuged at 3,200 g for 10–15 min, or until the entire volume had passed through the units. The Centricon units were then inverted, and the eluate was collected by centrifugation at 1,000 g for 1 min. A total of 350–1000 µl of eluate was collected per sample, from which RNA was extracted using QIAamp Viral RNA mini kit. In all RNA extractions, luciferase control RNA was added and tested as describe above. Two concentrated samples with the lowest Cq value were also analyzed under electron microscope using negative staining as described previously (Bačnik et al., 2020) and one by mechanical inoculation of four tomato plants cv. Moneymaker. Four weeks after mechanical inoculation, the infection of tomato plants on newly developed leaves was checked using M&W RT-qPCR.

### 2.4 Determination of the highest dilution of ToBRFV infected plant material still able to infect test plants

Tomato leaves (1 g) infected with ToBRFV were macerated in tap water (10 ml) using extraction bags with synthetic intermediate layer for filtration (Universal Extraction bags 12 × 15 cm, Bioreba, Switzerland) and tenfold dilutions were prepared using tap water as diluent. These dilutions were tested by LFD. RNA was extracted from the dilutions with QIAamp Viral RNA mini kit and tested using M&W RT-qPCR (Cq values for the obtained dilution series are provided in **Table 1**). Approximately 300-500 μl of each dilution of ToBRFV infected plant material were used for mechanical inoculation of four test plants of tomato. Two control plants were inoculated with non-infested tap water (confirming absence of ToBRFV with RT-qPCR). Symptom development on the test plants was monitored for up to nine weeks. Infection with ToBRFV on newly developed leaves was checked two weeks after inoculation with LFD and four and nine weeks after inoculation with M&W RT-qPCR (analysis was performed on RNA extracted from a pool of all four test plants together).

**Table 1:** Results of testing dilutions series of ToBRFV infested sap.

Dilution	ToBRFV infested water		Test plants^f^			
	LFD	RT-qPCR (Cq)^a^	Symptoms^b^	LFD^c^	RT-qPCR (Cq)^a,d^	RT-qPCR (Cq)^a,e^
10^-1^	+	4	+	+	NT	NT
10^-2^	+	7	+	+	NT	NT
10^-3^	+	10	+	+	NT	NT
10^-4^	+	13	+	+	NT	NT
10^-5^	+	17	+	+	NT	NT
10^-6^	+	20	+	+	NT	NT
10^-7^	–	22	–	–	NT	undet
10^-8^	–	25	–	+	13	NT
10^-9^	NT	25	–	–	NT	undet
10^-10^	NT	26	–	–	30	NT
10^-11^	NT	29	–	–	37	38
10^-12^	NT	33	–	–	NT	undet
10^-13^	NT	34	–	–	38	37
10^-14^	NT	35	NT	NT	NT	NT
NC	–	undet	–	–	37	undet

a The presence of ToBRFV RNA in water samples investigated by M&W RT-qPCR. The average Cq values of three replicates are given. Variation among technical replicates was ±0.5 from the mean Cq for Cq values <32 and ±0.7 from the mean Cq for Cq values ≥33.

b Symptoms observed were leaf curling, shoestring, bubbling and mosaic.

c Test plants were tested 2 weeks after mechanical inoculation with LFD.

d Test plants were tested 4 weeks after mechanical inoculation.

e Test plants were tested 9 weeks after mechanical inoculation.

f Each dilution was inoculated on 4 tomato plants, except NC, which was inoculated on 2 tomato plants.

+, Positive; -, Negative; Undet, No signal obtained with RT-qPCR; NT, not tested; NC, negative control.

### 2.5 Survival of the ToBRFV in an aqueous environment

**Figure 1:**
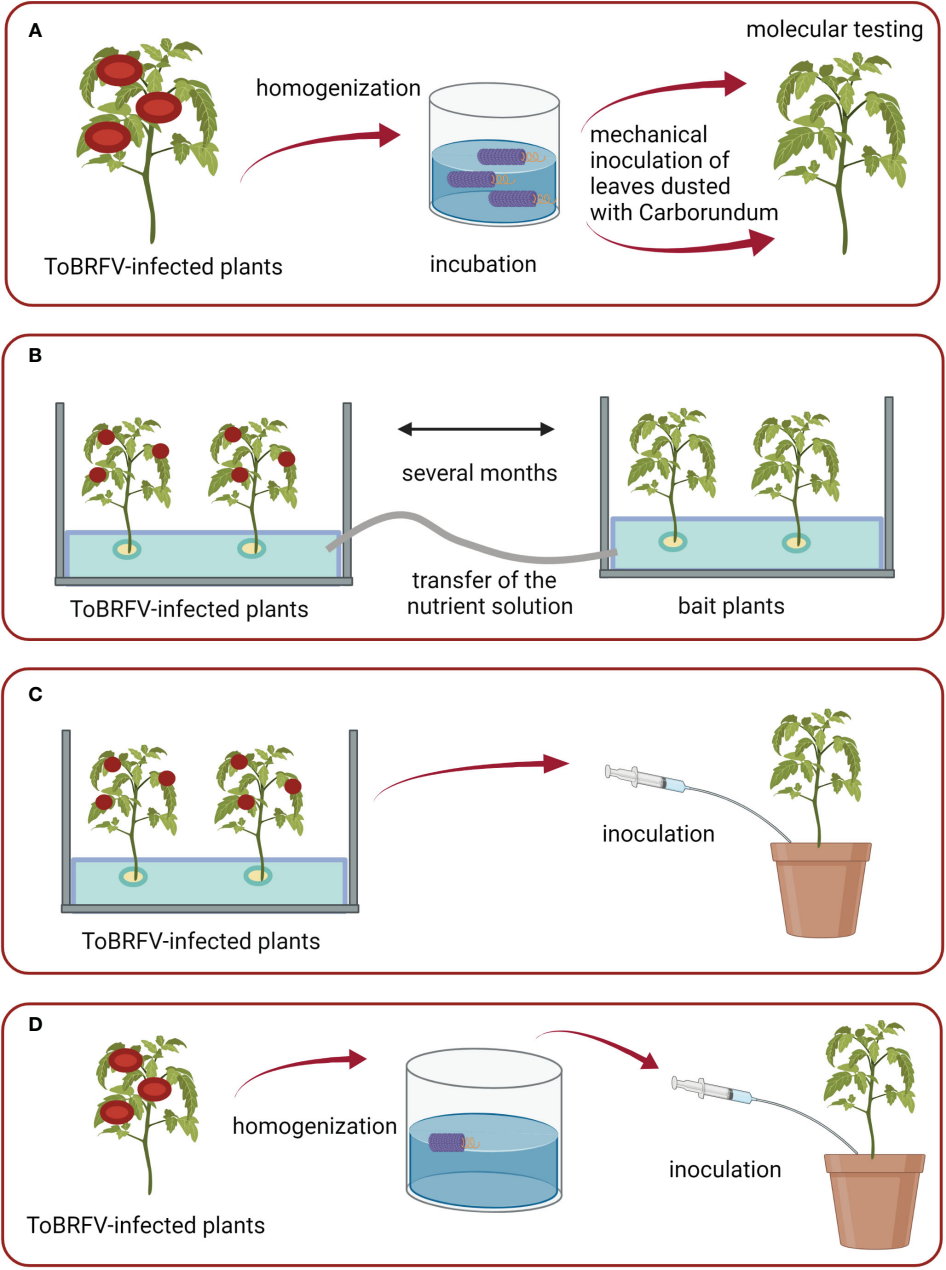
Schematic of conducted experiments: **(A)** Set-up of the study on the survival of ToBRFV in aqueous environment; **(B)** Study of water-mediated transmission in an experimental hydroponic system; **(C, D)** Studies on the transmission of ToBRFV to plants growing in substrate irrigated with infested water. Figures were created with BioRender.com.

With the aim of investigating how long ToBRFV can remain infectious in aqueous environments, the following studies were performed. The ToBRFV spike source was obtained as described in previous section and was then used to spike 1 L of tap water. Three dilutions of ToBRFV infected plant material in water were prepared: 10^-2^, 10^-4^, and 10^-6^. These dilutions of spiked infested water were stored in a quarantine greenhouse and sampled weekly. RNA from these water samples was extracted using QIAamp Viral RNA mini kit followed by M&W RT-qPCR analysis. ToBRFV infectivity in collected samples was determined *via* the mechanical inoculation of an aqueous solution onto four or five test plants of tomato (**Figure 1A**). Symptom development was monitored up to four weeks post inoculation and results were confirmed by M&W RT-qPCR or LFD analysis (analysis was performed on a pool of all four/five test plants together). Two control plants were included in the study each week, inoculated with non-infested tap water (confirming absence of ToBRFV with RT-qPCR).

### 2.6 Water-mediated transmission in experimental hydroponic systems

Three separate experimental hydroponic systems were designed to study the possibility of water-mediated transmission of ToBRFV. In each, six ToBRFV-infected tomato plants cv. Moneymaker were placed in a glass tank (dimensions: 0.6 × 0.4 × 0.4 m) filled with nutrient solution (Johnson et al., 1994). Few weeks later, healthy (bait) plants of tomato cv. Moneymaker were placed into separate tanks as two-week-old seedlings (approximately 5-10 cm high). In each of these tanks, six bait plants were placed. Before the plants were placed into the tanks, the substrate was washed away from the roots with water. In each tank, the plants were grown in 10 cm-diameter plastic pots filled with stone-wool substrate (Grotop Master Dry, Grodan, the Netherlands). The bait plants were irrigated with the nutrient solution from the tank with the inoculated plants. Two separate tanks of bait plants were used for the first and third experiments, while only one tank of bait plants was used for the second experiment.

Special care was taken to prevent any contact between the mechanically inoculated plants and the bait plants, and between the nutrient solution from the inoculated plants and the upper green parts of the bait plants. Styrofoam (thickness, 3 cm; positioned in the tanks approximately 5 cm above the bottom) was used keep the upper green parts separate from the root parts and the nutrient solution. During the first experimental period, infected plants and bait plants were grown in the same chamber of the greenhouse, and nutrient solution was pumped directly from the tanks containing inoculated plants to the root zones of the tanks containing bait plants using pumps and plastic tubes (Statuary fountain pump PondoCompact 300, Pontec, Germany) (**Figure 1B**). During the second and third experimental periods, infected plants and bait plants were grown in separate chambers of the greenhouse, and nutrient solutions were transferred from the tanks containing inoculated plants to the root zones of the tanks containing bait plants using a bottle and glass funnel. Occasionally, the roots of inoculated plants and bait plants of first and second experiments were gently stirred by hand to imitate the real conditions in a hydroponic system, where injury to root systems is expected due to the presence of macrobiota and growth of roots through glass wool. The lower parts of the tanks were wrapped with aluminum foil, to prevent algal growth in the nutrient solution.

Both nutrient solution and leaves and root tissue of inoculated and bait plants were sampled at regular intervals. RNA was extracted from both nutrient solution samples using QIAamp Viral RNA minikit and from plant tissue samples (pools from six plants) using RNeasy Plant mini kit. All extracted RNA was analyzed using M&W RT-qPCR. In addition, the infectivity of ToBRFV in the nutrient solution was checked with mechanical inoculation of tomato plants as described above. Several control plants of tomato grown in substrate at the same time in the same greenhouse, and fresh nutrient solutions were also tested.

### 2.7 Transmission of ToBRFV through injection of infested water into the substrate

Ten-day-old seedlings (about 5–10 cm tall) of tomato plants cv. Moneymaker were each planted in 18 cm diameter plastic pots with the substrate (Fruhstorfer Erde Aussaat und Stecklingserde, Hawita, Germany). For the first three weeks, 50 ml of infested water/nutrient solution was added to the substrate in each pot once a week using a syringe, and then 100–150 ml of infested water/nutrient solution was added per pot two to three times a week depending on the growth stage of plants. Precautionary measures were taken to prevent contact of green parts of the bait plants with the exterior of syringe and infested water/nutrient solution. The nutrient solution from the tank of inoculated plants from the second experiment described above was injected into the substrate of six pots (**Figure 1C**), while freshly prepared ToBRFV-infested tap water was injected into the substrate of the other four pots (**Figure 1D**). This ToBRFV-infested water was prepared each week by macerating infected tomato leaves in tap water in extraction bags (Universal Extraction Bags, Bioreba, Switzerland). Control plants watered with the same amount of non-infested nutrient solution/tap water were also included. Each week, RNA extracted from a pool of upper leaves of all six/four plants per experiment and from a pool of control plants were analyzed by M&W RT-qPCR.

### 2.8 Analyses of the water from commercial tomato greenhouses

Tomato commercial greenhouses from different countries from north-west Europe were used to monitor the presence of ToBRFV. Greenhouses with (Greenhouse D) or without (Greenhouse A, B, C) previous ToBRFV outbreaks were chosen and drain water samples were gathered from a collection point before disinfection in the greenhouse water circulation system. RNA was extracted from the samples using the RNeasy Plus kit (Qiagen, USA) and the samples were tested using ISF-ISHI-Veg RT-qPCR.

To monitor the correlation of ToBRFV building up in water with the development of infection in plants, leaf samples from the young leaves at the top of the plant and from the sepals were also taken. RNA from collected samples was extracted and analyzed using the same procedure as for water samples.

The infectivity of ToBRFV in drain water was assessed using bio-assay in a growth chamber. Five tomato plants of cv. Climbo of 10 days old were planted in styrofoam pots (480 ml) containing a soil mixture suitable for young plants (DCM potting mix for sowing and cutting). For the duration of the experiment, the plants were watered three times a week with 50 ml of ToBRFV-infested drain water collected from a grower with an active ToBRFV outbreak. Irrigation with ToBRFV-infested water was carried out for four weeks, and in the meantime the water was stored at 4°C. Before the treatment, RNA extracted from the infested water sample was tested with ISF-ISHI-Veg RT-qPCR. Additionally, five tomato plants of cv. Climbo of 10 days old were first dusted with Carborundum and then inoculated on the cotyledons with the same ToBRFV-infested drain water sample. Symptom development was monitored for up to four weeks after the start of the treatment and confirmed by ISF-ISHI-veg RT-qPCR analysis of extracted RNA from upper newly developed leaves. As a positive control, five plants were mechanically inoculated with ToBRFV-infected leaf material. The inoculum was prepared by homogenizing 1 g of frozen ToBRFV-infected leaf material (-20°C) in 3 ml phosphate buffer (pH 7.4) using metallic beads. As a negative control, five plants were inoculated with the phosphate buffer. The experiment was performed in a growth chamber under controlled conditions (25 ± 2°C during the light period (14 h) and 19 ± 2°C during the dark period (10 h)).

In addition, genomic and coat protein degradation in drain water over time was assessed. A drain water sample of 2 L was collected from a grower with an active ToBRFV outbreak. Subsequently, 1 L of the sample was stored at 4°C and 1 L of the sample was stored at room temperature to monitor the integrity of the ToBRFV particles in drain water over time. Both samples were sub-sampled every two weeks. After RNA extraction using an RNeasy Plus kit, the water samples were tested with ISF-ISHI-Veg RT-qPCR and a near-full genome PCR (hereafter NFG-PCR) to determine the integrity of the RNA genome. The cDNA for the PCR was created using the qPCRBIO cDNA synthesis kit (PCR biosystems) with viral RNA as a template. The RT reaction was performed using the F-22 and F-6392 primers published by Eldan et al. (2022). Following synthesis, the viral cDNA was then amplified using the Herculase II fusion DNA polymerase (Agilent) using the same primers as for the RT reaction (Eldan et al., 2022). Additionally, the water samples were tested with DAS-ELISA (Agdia, Reagent set for ToBRFV) to determine the presence of the ToBRFV capsid protein.

## 3 Results

### 3.1 Detection of ToBRFV in Slovenian environmental water samples

The first detection of ToBRFV RNA in Slovenia was done in a wastewater sample from 2017 using high-throughput sequencing (Bačnik et al., 2020). Later, its RNA was detected by RT-qPCR in samples from a river in central Slovenia (one ToBRFV-positive sample from 2019 and one from 2020; both collected at the same location) and in samples from rivers and a pond used for crop irrigation from south-western Slovenia (one sample in 2019 and two samples in 2020; all collected at different locations, once in a pond and twice in a river), from south-eastern Slovenia (one sample from 2020; source: river) and from north-eastern Slovenia (one sample from 2021; source: unknown) (**Supplementary Table 1**). The concentration of ToBRFV RNA in these eight water samples is estimated to be low because a concentration step of the water samples was required for reliable detection; even after the concentration step, relatively high Cq values were obtained with RT-qPCRs: between 28 and 30; in the case of a concentrated water sample from 2021, the Cq value of the M&W RT-qPCR was 32. The presence of ToBRFV RNA in this particular sample was additionally confirmed by sequencing the PCR and nested-PCR products (data not shown). M&W RT-qPCR or ISF-ISHI-Veg RT-qPCR for other water samples from 2019–2022 revealed either no signals or Cq values above 34.

### 3.2 Comparison of different approaches for the detection of ToBRFV RNA in water

Centricons were found to be efficient for concentrating ToBRFV from infested nutrient solution samples stored at 4°C for six months, as analysis of RNA from concentrated nutrient solution samples by RT-qPCR yielded values up to 8 Cq lower than analysis of RNA from nonconcentrated nutrient solution samples (**Table 2**). The concentration step proved unnecessary for samples in which high virus concentrations were expected. In such cases, late signals (high Cq values) were obtained even when samples were analyzed directly, i.e., without RNA extraction. In addition, it was found that the extracted RNA from concentrated and non-concentrated samples of the nutrient solution from the tray containing healthy plants also gave some late signals in RT-qPCR, most likely due to contamination from growing these plants in the same chamber as the ToBRFV-infected plants.

**Table 2:** Results of RT-qPCR analyses of nutrient solution exposed to ToBRFV-infected plants.

No. of days^a^	Three infected plants per tray^b^			One infected plant and two healthy per tray^b^			Three healthy plants per tray^b^		
	D	E	C+E	D	E	C+E	D	E	C+E
2	Undet	35	35	Undet	37	Undet	Undet	36	36
5	Undet	36	30	Undet	40	Undet	Undet	36	Undet
7	Undet	34	28	Undet	Undet	36	Undet	37	37
9	35	30	24	34	28	25	Undet	36	Undet
12	35	23	17	35	24	20	Undet	35	32
14	36	23	17	36	22	15	Undet	34	31
16	33	19	15	33	25	18	Undet	32	34
19	33	22	15	33	23	15	Undet	34	28

a Days after exposure of the nutrient solution to the ToBRFV-infected plants.

b The nutrient solution was analyzed by M&W RT-qPCR in three different ways: D, directly without RNA extraction; E, RNA extracted; C+E, RNA extracted from the concentrated sample. The average Cq values of three replicates are given. Variation among technical replicates was ±0.7 from the mean Cq. Undet, No signal obtained with RT-qPCR.

The concentrated samples of nutrient solution with the estimated highest virus concentration (two samples with Cq 15 from the tray with three infected plants) were further examined by electron microscopy and one of these two also by mechanical inoculation of test plants. No virus particles were observed in these two samples under the electron microscope, and the virus did not replicate in test plants.

### 3.3 Determination of the highest dilution of ToBRFV infected plant material still able to infect test plants

The tests of dilution of ToBRFV infected plant material in water confirm that a higher viral load is required to detect the virus with LFD and by inoculation of test plants than when using RT-qPCR (**Table 1**). With test plants assays and LFD, the presence of infectious ToBRFV was confirmed in all samples with Cq ≤ 20 by M&W RT-qPCR. However, in one example, infection of plants was observed at an even higher dilution (10^-8^, Cq = 25), indicating that sporadic transmission to test plants from water samples with higher Cq is possible, although accidental transmission during the course of the experiment cannot be completely ruled out, although the results of controls were negative.

### 3.4 Survival of ToBRFV in aqueous environment

Infected leaves of test plants were macerated and incubated in water under quarantine conditions in the greenhouse. ToBRFV remained infectious for up to four weeks in water spiked with ToBRFV at dilutions of 10^-2^ and 10^-4^, and only one week in water spiked with ToBRFV at a dilution 10^-6^ (**Table 3**). In all cases, ToBRFV RNA was detected for much longer, at least 15 weeks after preparation of the infested water (**Table 3** shows data only up to Week 9).

**Table 3:** Detection of ToBRFV in artificially infested water stored in quarantine greenhouse.

Time (weeks)^a^	ToBRFV 10^-2^ dilution		ToBRFV 10^-4^ dilution		ToBRFV 10^-6^ dilution	
	RT-qPCR (Cq)^b^	Test plants^c^	RT-qPCR (Cq)^b^	Test plants^c^	RT-qPCR (Cq)^b^	Test plants^c^
0	7	**+**	14	**+**	21	**+**
1	7	**+**	14	**+**	21	**+**
2	7	**+**	15	**+**	22	–
3	7	**+**	16	–	23	–
4	8	**+**	15	**+**	27	–
5	10	–	21	–	26	–
6	10	–	20	–	21	–
7	10	–	21	–	22	–
8	12	–	21	–	22	–
9	11	–	21	–	21	–

a Weeks after water inoculum was prepared.

b The presence of ToBRFV RNA in water samples investigated by M&W RT-qPCR. The average Cq values of three replicates are given. Variation among technical replicates was ±0.5 from the mean Cq.

c Infectivity in water was monitored by observing the development of symptoms on inoculated test plants (for each time point and for each ToBRFV dilution 4 to 5 test plants were used) along with RT-qPCR or LFD analysis. If no symptoms developed on test plants 4 weeks after mechanical inoculation, the absence of ToBRFV was confirmed by RT-qPCR. +, Positive (symptoms and LFD positive or Cq less than 10); -, Negative (no symptoms and ToBRFV presence not confirmed by RT-qPCR). Negative controls were always negative.

### 3.5 Water-mediated transmission of ToBRFV in hydroponic and substrate systems

**Figure 2:**
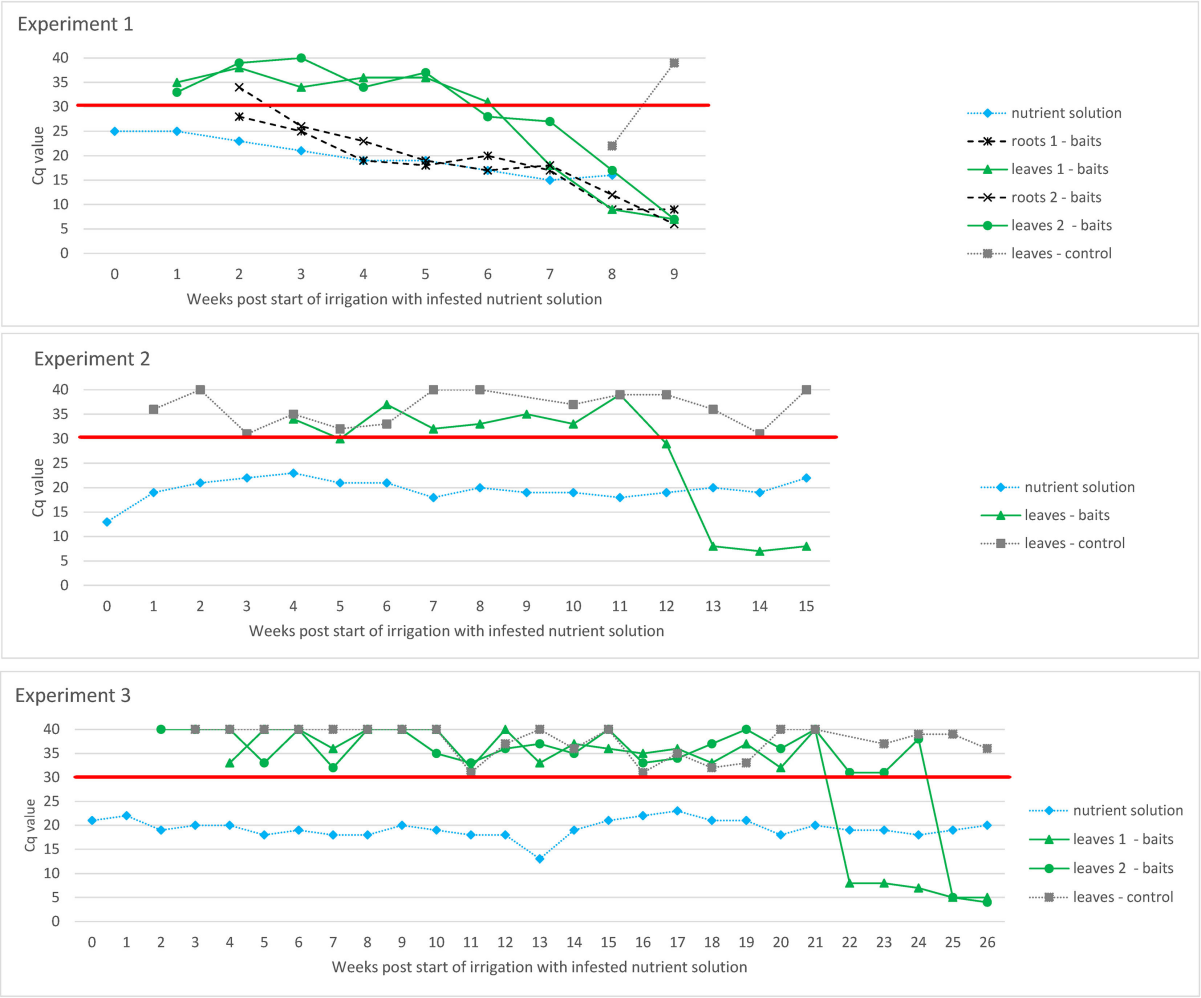
Evaluation of ToBRFV infection in tomato plants grown in an experimental hydroponic system with ToBRFV-infested nutrient solution in three separate experiments. Y-axes show Cq values from M&W RT-qPCR. Plotted Cq value represent average of three technical replicates. Variation among technical replicates was ±0.5 from the mean Cq for Cq values <32 and ±0.7 from the mean Cq for Cq values ≥33. The X-axes show the weeks after the start of irrigation with infested nutrient solution. The black signs and lines designate the results of the leaf and roots samples of bait plants. The results of the examination of the nutrient solution, the leaves of the control plants and, in the case of Experiment 1, the results of the root samples of the bait plants are shown in grey. The red line shows the Cq value of 30 below which the result was considered positive.

ToBRFV RNA was detected in the nutrient solution as early as the first week after infected plants were placed in a glass tank (Cq value of M&W RT-qPCR for the nutrient solution was 25, whereas the Cq value of infected leaves was 7; data not shown). In each experiment, six tomato plants were placed in the tanks with bait plants. Experiments 1 and 2 had two replicas. Five, seven, and eight weeks after the start of the experiment, the Cq values of M&W RT-qPCR of this nutrient solution were 19, 15, and 16, respectively, and the ToBRFV in the nutrient solution proved infectious at all these time points *via* the mechanical inoculation of test plants (data not shown). In addition, three separate experiments examined infection *via* the roots of tomato plants grown in other glass tanks in which only the roots were exposed to ToBRFV-infested water (i.e., bait plants; **Figure 2**). In the first experiment, symptoms such as leaf curling, shoestring, blistering, and mosaic were observed on the leaves of bait plants in one tank eight weeks and in the other tank nine weeks after the start of irrigation with the infested nutrient solution. This correlated with the decrease in RT-qPCR Cq value below 10 for root and leaf samples of the bait plants. Also, in the second and third experiments, the symptoms observed on the bait plants correlate with the drop in RT-qPCR Cq value below 10 for the leaf samples (root samples were not tested). However, in the second experiment, this was observed 13 weeks after beginning irrigation with the infested nutrient solution, and in the third experiment, in which roots were not occasionally stirred by hand as in the first two experiments, this was observed even later (between five and six months after irrigation with the infested nutrient solution began). The Cq values of the leaves of control plants grown in the same chamber of the quarantine greenhouse as the bait plants were above 30, except at Week 8 in the period of Experiment 1, when the Cq value of the control plants was 22. However, this appears to be due to adventitious spread rather than infection, as later tests on the same plants showed a much higher Cq value and, in addition, the control plants did not show typical virus-like symptoms.

**Figure 3:**
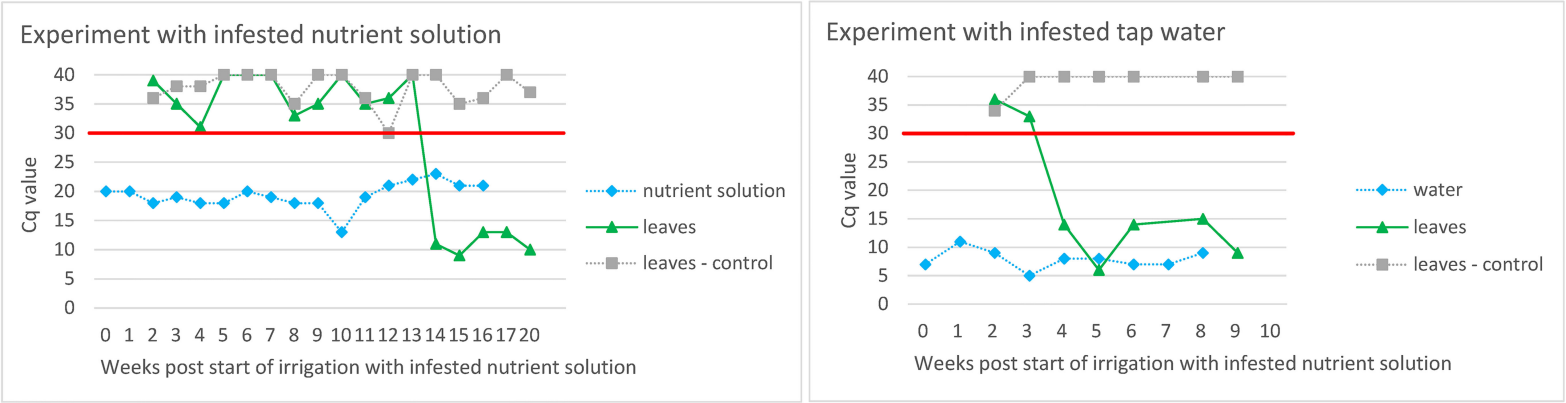
Evaluation of ToBRFV infection in tomato plants grown in substrate irrigated with ToBRFV-infested nutrient solution/tap water. The Y-axes show Cq values from M&W RT-qPCR. Plotted Cq value represent average of three technical replicates. Variation among technical replicates was ±0.5 from the mean Cq for Cq values <32 and ±0.7 from the mean Cq for Cq values ≥33. The X-axes show the weeks after the start of irrigation with infested nutrient solution/tap water. The black symbols and lines designate the results of the leaf samples of tomato plants grown in substrate watered with infested nutrient solution/tap water. The results of the examination of the nutrient solution and the leaves of the control plants are shown in grey. The red line shows the Cq value of 30 below which the result was considered positive.

Similar to the experiments described above, ToBRFV was also detected in leaves of tomato plants grown in a substrate that had been irrigated with ToBRFV-infested nutrient solution and with ToBRFV-infested tap water (**Figure 3**). The virus detection signal strength in infested tap water was lower than in the nutrient solution, and plants irrigated with infested tap water became infected with ToBRFV earlier than plants irrigated with infested nutrient solution.

### 3.6 Results of ToBRFV monitoring in drain water from commercial tomato greenhouses

**Figure 4:**
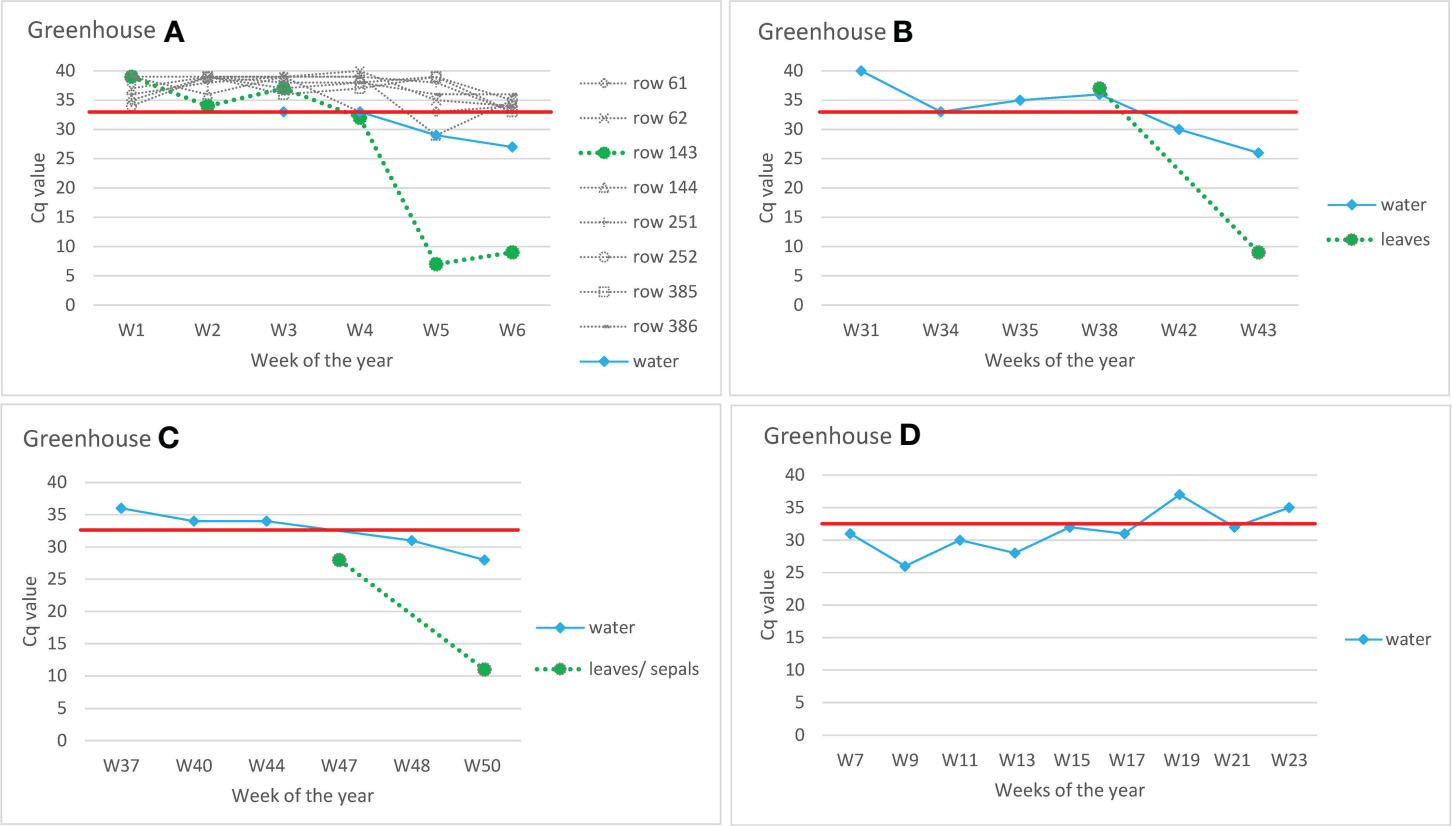
Detection of ToBRFV in recirculating drain water from commercial tomato greenhouses. Y-axes show Cq values of CSP1325 primers and probe of ISF-ISHI-Veg RT-qPCR. Similar Cq values were also obtained with CaTa28 primers and probe of ISF-ISHI-Veg RT-qPCR (data not shown). The X-axes show the week number at the time the samples were taken for analysis. The black dots are the results of the water samples, the grey dots are the results of the leaf samples. Greenhouse **(A)** pooled samples of leaves from different rows (row 61, row 62, etc.) and drain water samples were analyzed weekly to monitor for a possible outbreak of ToBRFV. Plants from row 143 were symptomatic at Week 6 (W6). Greenhouses **(B, C)** drain water samples were analyzed at six/five selected time points; leaf samples were analyzed at the time when symptoms first appeared: W43 in the case of Greenhouse **(B)** and W50 in the case of Greenhouse **(C)** In addition, leaf sample of W38 was analyzed in Greenhouse B and sepal sample of W47 in Greenhouse **(C)** Greenhouse **(D)** example of testing drain water in greenhouse in the season following a ToBRFV outbreak; no symptoms appeared in the new crop, and random sampling of plants by authorities did not detect the virus. The red line shows the Cq value of 32.5 below which the result was considered positive.

In Greenhouse A, eight rows were monitored weekly for a possible outbreak of ToBRFV, by randomly collecting pooled leaves of different tomato plants spread randomly across the row. In Week 5, a sudden drop in ToBRFV Cq value was measured in one row (143) (**Figure 4**). Symptoms were monitored in all plants from greenhouse A, and the first symptomatic plants were observed at week 6. It is notable that in Week 5 (i.e., one week before symptoms were observed), the Cq value for ToBRFV from the drain water dropped below 30. Similarly, in Greenhouses B and C, the decreasing Cq values of the drain water samples indicate a possible propagation of the virus in the tomatoes before the first symptoms were observed (in Greenhouse B, symptoms were observed at Week 43, and in Greenhouse C at Week 50; in both cases, the plants already showed low Cq values at this time). In practice, however, it is possible that a residue (not necessarily an infectious virus) of ToBRFV is detected. This is illustrated by an example from commercial Greenhouse D in the season following a ToBRFV outbreak. In this greenhouse, where no symptoms were observed on new tomato plants and where authorities also did not detect the virus during random sampling of plants throughout the greenhouse, the ToBRFV concentration in the drain water gradually decreases (shown as increasing of Cq values of ISF-ISHI-Veg RT-qPCR) over time.

Using drain water from a commercial grower with a ToBRFV outbreak, it was possible to infect plants *via* mechanical inoculation of leaves (three of five mechanically inoculated test plants became infected); however, this did not work *via* roots (none of the plants irrigated with this drain water for four weeks became infected) (**Table 4**). The drain water sample that was used to inoculate the plants had a Cq-value of 16. Finally, the results which followed the degradation of ToBRFV viral particles in drain water stored at 4°C showed that ToBRFV RNA in drain water can be detected using RT-qPCR and by NFG-PCR for at least fourteen weeks; however, clear detection of the viral coat protein by ELISA was possible for eight weeks, and only a weak signal was obtained at weeks 12 and 14 (**Table 5**). Similarly, the detection of viral RNA in drain water stored at room temperature was possible for at least fourteen weeks, but the detection of viral coat protein in water stored at room temperature was not possible even at Week 2 (**Table 5**).

**Table 4:** Results of analyzing test plants 4 weeks after mechanical inoculation and 4 weeks after the start of irrigation with drain water from the greenhouse with ToBRFV outbreak.

Replicate	Watering with ToBRFV infested water	Mechanical inoculation of leaves		
		ToBRFV infested water	Positive control	Negative control
1	40 ^a^	39 ^a^	6 ^a^	37 ^a^
2	37	11	6	Undet
3	36	7	6	40
4	38	9	6	Undet
5	37	32	6	Undet

a The Cq values of CSP1325 primers and probe of ISF-ISHI-Veg RT-qPCR are given. Similar Cq values were also obtained with CaTa28 primers and probe of ISF-ISHI-Veg RT-qPCR (data not shown). Undet, No signal obtained with RT-qPCR. Symptoms were observed on all test plants that had a Cq value below 12.

**Table 5:** Results of the analysis of drain water from the greenhouse with ToBRFV outbreak after storage at room temperature and at 4^°^C.

No. of weeks	Room temperature			4^°^C		
	RT-qPCR^a^	ELISA	NFG-PCR	RT-qPCR^a^	ELISA	NFG-PCR
0	16	**+**	**+**	16	**+**	**+**
2	17	-	**+**	17	**+**	**+**
4	16	-	**+**	14	**+**	**+**
8	16	-	**+**	17	**+**	**+**
10	16	–	+	15	–	+
12	14	–	+	14	+^b^	+
14	15	–	+	13	+^b^	+

a The Cq values of CSP1325 primers and probe of ISF-ISHI-Veg RT-qPCR are given. Similar Cq values were also obtained with CaTa28 primers and probe of ISF-ISHI-Veg RT-qPCR (data not shown).

b Weak positive result. +, Positive; -, Negative. The negative controls included in each run of each method were always negative.

## 4 Discussion

In Slovenia, ToBRFV RNA was detected in a wastewater sample from 2017 (Bačnik et al., 2020) and then in samples from a river and in samples from rivers and a pond used for crop irrigation in different parts of Slovenia (**Supplementary Table 1**) before the ToBRFV-infected plants were found (Vučurović et al., 2022). None of the locations where ToBRFV-contaminated water was sampled is close to the location of that finding of ToBRFV-infected plants (Vučurović et al., 2022), which is also the only finding of ToBRFV-infected plants in Slovenia to date. Therefore, the source of water contamination with ToBRFV in Slovenia remains unknown. Many other studies indicated that tobamoviruses, including ToBRFV can be found in human gut and oropharynx (Aguado-García et al., 2020) or in wastewater samples at high relative abundance (Rothman et al., 2021). This opens up the possibility that the origin of the ToBRFV sequences in Slovenian wastewater may be faecal contamination rather than agricultural runoff but based on the available data it is not possible to draw any conclusions. However, the source of ToBRFV contamination of drain water in commercial tomato greenhouses from north-west Europe was clearly associated with the infected tomato plants growing in these greenhouses.

As described in Mehle and Ravnikar (2012), potential sources of plant viruses in environmental waters include roots of infected plants growing in an ecological niche near the water, injured or decaying plant material, and sewage. Some plant viruses present in vegetables or fruits may pass through the digestive tract (Zhang et al., 2006) and be released into wastewater that could find its way into environmental waters, or surface wash-out of locally scattered and infected decaying plant debris and associated soil surface layers, including animal feces, virus-containing seeds, etc., could bring the plant viruses into waters (Mehle et al., 2018). Under experimental conditions, we have shown that infectious particles of ToBRFV can be released from tomato roots of ToBRFV-infected plants into nutrient solution (**Figure 2**). The release of viruses from roots into water has already been demonstrated for some other plant viruses, such as tobacco necrosis virus (TNV), tobacco mosaic virus (TMV), PepMV and PVY, as well as for PSTVd (Yarwood, 1960; Schwarz et al., 2010; Mehle et al., 2014).

The ToBRFV concentration in Slovenian water samples was below the detection limit of serological testing (LFD) and mechanical inoculation of test plants, so it is not known whether the detected ToBRFV in Slovenian waters was present as infectious particles, as non-infectious particles, or only as RNA. However, the data presented here shows that in case of an active ToBRFV outbreak in commercial greenhouses, a much higher ToBRFV concentration can be reached in the drain water, and by mechanical inoculation of test plants it was confirmed that the ToBRFV particles detected in the water are infectious (**Table 4**). In addition, the data presented here demonstrate that under experimental conditions ToBRFV remains infectious in water at room temperature for up to four weeks (**Table 3**). There are also some data on the survival of other plant viruses in water and nutrient solutions under greenhouse conditions (Mehle et al., 2018). For example, PVY has been shown to remain infectious in aqueous environments for up to one week, PepMV for up to three weeks, and tomato mosaic virus (ToMV) for at least six months (Pares et al., 1992; Mehle et al., 2014). The differences in survival observed between these viruses are likely due to their different structures and to different experimental conditions (Mehle et al., 2018). For example, the survival time of PVY in water has been shown to be much longer (up to 10 weeks) when stored at 4°C, likely due to the higher stability of the coat protein at lower temperatures (Mehle et al., 2014). Correspondingly, the coat protein of ToBRFV was detected in a drain water sample stored at 4°C even after fourteen weeks by ELISA, whereas this was no longer possible after two weeks when the sample was stored at room temperature (**Table 5**).

The determined survival time in aqueous environments also depends on the virus concentration in the water, and this was demonstrated by including different dilutions of ToBRFV infected plant material in the survival experiments (**Table 3**). In addition, several other factors may influence these results, such as the susceptibility of the test plants used, the presence of clay particles or organic matter that may protect plant viruses from inactivation in water (Mehle et al., 2018). In the case of the experiment with ToBRFV 10^-4^ dilution, infectivity in water three weeks after water inoculation could not be confirmed, while four weeks after water inoculation, transmission to test plants by mechanical inoculation was successful (**Table 3**). The reason for this unexpected result could be a combination of the low number of infected virions present and the limited number of test plants per time point and/or the different susceptibility of the test plants (although we used test plants of the same batch, the plants differ slightly in growth stage at the time of inoculation).

The presence of plant viruses in waters may have epidemiological importance if the viruses can enter plants through the roots or through the upper parts of plants when contaminated water is used for irrigation (Mehle et al., 2018). Our experimental data show that ToBRFV from the nutrient solution or irrigation water can infect healthy tomato plants through the roots and eventually spread to the upper parts of the plants, where it can be detected after one to six months (**Figure 2**). This study demonstrates that the time required for symptom development and reliable detection of ToBRFV in the upper green parts of tomato plants depends on the virus concentration in the water as well as the severity of root damage. Under conditions expected in production systems or in nature, root systems are damaged by the presence of macrobiota and root growth through soil or glass wool. In two of three experiments mimicking the hydroponic system, we damaged the roots by lightly stirring them by hand, and in these two experiments, infection of the plants was observed earlier (similar to the experiment in which the plants were grown in soil) than in the experiment in which the roots were not additionally damaged and in which only stirring with the water flow generated by the work of the pump was used to mix the nutrient solution. Our results show that infection occurred even when tomato roots were not severely damaged but were exposed to moderate concentrations of ToBRFV in the nutrient solution (about 20 Cq), probably due to the high infectivity of ToBRFV.

For a reliable assessment of the role of water as a route of spread for the virus, it is important to conduct long-term experiments because in practice, when recycled water is used for irrigation, tomato plants can be inoculated repeatedly throughout the growing season, which can take 10 months (Mehle et al., 2014). For example, for PSTVd, in the short-term experiment (repeated addition of inoculum to the rooting substrate of tomato for up to 10 consecutive days) by Verhoeven et al. (2010), transmission through the roots was not confirmed, whereas this was confirmed in the long-term experiment (several months) by Mehle et al. (2014). However, due to technical limitations, it was only possible to conduct a short-term study of ToBRFV transmission using drain water from a commercial tomato greenhouse with an active ToBRFV outbreak (**Table 4**). This was done by repeatedly adding the drain water to the rooting substrate of the tomato over a period of only four weeks, and therefore it is not surprising that the plant did not show ToBRFV transmission in this experiment. In addition, drain water used in this study was not taken fresh but stored at 4°C, which could also affect the final result.

Waterborne transmission of some other plant viruses has also been confirmed (Mehle and Ravnikar, 2012; Mehle et al., 2014). As discussed in Mehle et al. (2018) for other waterborne viruses, this is likely not the most efficient route of virus transmission for ToBRFV either. Considering that ToBRFV can quickly and effectively spread mechanically to neighboring plants, the possibility that it can also be transmitted by water should be considered an important issue. This is especially important to consider in hydroponic systems or other systems in which recycled water is used, and in such cases our data show that it is worth monitoring the water for ToBRFV to predict critical locations and moments for viral disease onset. In addition, the data presented in this study shows that an outbreak of ToBRFV can be detected with regular monitoring of drain water. Using the example of a commercial tomato greenhouse (Greenhouse A in **Figure 4**), it is clear that monitoring drain water is more efficient than analyzing the large number of plant samples that must be taken in a greenhouse for early detection and screening purposes. Although the RNA of ToBRFV is detectable by PCR-based methods long after the virus has lost its infectivity, long-term monitoring can reveal changes in the amount of virus in water by qPCR, which could be used to monitor ongoing infections.

Viruses are usually present in water at very low concentrations but can still pose a significant health risk, because very low titers are often required for infection (Mehle et al., 2018). Our data suggest that monitoring large bodies of water, where ToBRFV may be highly diluted, requires an appropriate concentration step. Concentration of ToBRFV from water samples using CIM monolithic chromatography proved to be efficient but requires complex equipment that is not available in many laboratories. For this reason, ultrafiltration with Centricon Plus-70 Centrifugal Filter Units, which can be used in any laboratory with a benchtop centrifuge, was evaluated and found to also be very efficient for concentrating ToBRFV from water samples (**Table 2**). However, it has yet to be determined whether the use of a lower initial sample volume in Centricon units (up to 100 ml) compared to CIM monoliths (up to 5 l) has an adverse effect on the sensitivity of the final detection when used in large water bodies. For the moment, for analysis of cleaner water sources (river, tap, underground) analyzing larger volumes (CIM monoliths) would be recommended, while for waters with a higher contamination burden, e.g., hydroponics, wastewater, recirculating water, lower volumes (Centricon) may suffice. The other limitation of the experiment comparing different approaches for the detection of ToBRFV in water (direct analysis of water samples with RT-qPCR, analysis of extracted RNA, and analysis of RNA from the concentrated water samples) is that six-month-old water samples were used. This means that although the water samples were stored at 4°C, our tests most likely detected ToBRFV RNA and not virus particles. This is also indicated by the unsuccessful transfer of ToBRFV from a concentrated water sample to test plants and the fact that no virus particles were found by electron microscopy in two concentrated water samples examined. Nevertheless, it confirms once again the resilience of the ToBRFV RNA detection by RT-qPCR which can persist and stay infective for several months on some glasshouse surfaces (Skelton et al., 2021).

The main problem faced in performing all these experiments was the adventitious spread of ToBRFV signal in the greenhouse. Using RT-qPCRs, high Cq values were obtained (between 30 and 40) in many control plants, in water samples, and in swabs from walls that were never in direct contact with contaminated water or with infected plants, but were from the same chamber as heavily infected plants or heavily contaminated water (data not shown). In one case, ToBRFV was detected in collected control plants with a Cq value of 22, but a week later the analysis of the same plants resulted in a much higher Cq value. Since there was no significant drop in Cq values in these samples over periods from two to six months, and no symptoms were seen in the negative control plants, it is assumed that these were environmental contaminations that never led to real infection. However, this is very worrying as such low signals may also mean low virus titer, which theoretically could lead to infection in individual cases. Therefore, this should be investigated in further studies, because the situation found under experimental conditions is also likely to be the situation in commercial greenhouses during and after ToBRFV outbreaks as shown by Loh et al. (2022).

The results of our studies have shown that water can be an important source of ToBRFV inoculum under the experimental conditions. Potentially, such a scenario in intensive production can lead to significant yield losses in tomato production. Therefore, the results of this study are very important for the introduction of new water monitoring systems that can be used for larger scale studies. An improved, more affordable water monitoring system can play an important role in controlling ToBRFV and other tobamoviruses.

## Data availability statement

The original contributions presented in the study are included in the article/**Supplementary Material**. Further inquiries can be directed to the corresponding author.

## Author contributions

NM participated in the design of the study, drafting of the manuscript, and supervised with AV the conduct of the hydroponic transmission, soil transmission, and survival study experiments, conducted by MK, AV, IB, and JB. OMCF, IG-A, DK, JB, AV, and NM conceived and performed the experiments ToBRFV detection in various environmental waters. OMCF, AF, YL, IG-A, DK, KB, and NM planned and performed the experiments for testing the different approaches for ToBRFV RNA detection in water. EV and CV planned and performed the experiments related to commercial greenhouses. All authors contributed to writing and reviewing the manuscript and approved the submitted version.
