# Supplementary material for: Tomato brown rugose fruit virus in aqueous environments – survival and significance of water-mediated transmission
Source: Front Plant Sci. 2023 Jun 2;14:1187920. doi: 10.3389/fpls.2023.1187920 (PMC10275568; doi:10.3389/fpls.2023.1187920)
Supplement: Supplementary file 1 [file Table_1.docx]

Supplementary Material

Tomato brown rugose fruit virus in aqueous environments – survival and significance of water-mediated transmission

Nataša Mehle,^*^ Katarina Bačnik, Irena Bajde, Jakob Brodarič, Adrian Fox, Ion Gutiérrez-Aguirre, Miha Kitek, Denis Kutnjak, Yue Lin Loh, Olivera Maksimović Carvalho Ferreira, Maja Ravnikar, Elise Vogel, Christine Vos, Ana Vučurović

*** Correspondence:** Nataša Mehle: natasa.mehle@nib.si;

Supplementary Table 1. Origin of water samples and results of ToBRFV analyses in concentrated samples

| Sample designation | Type of sample | Source | Location | Collection year | Cq before concentration^a^ | Cq after concentration^a^ |
| --- | --- | --- | --- | --- | --- | --- |
| **Inf17-Linb** | **Wastewater** | **Influent** | **Central Slovenia location 1** | **2017** | **NT** | **30^b^** |
| **IN19SI02** | **Area** | **River** | **Central Slovenia location 2** | **2019** | **NT** | **30** |
| IN19SI06 | Area | Pond | South-west Slovenia location 1 | 2019 | NT | 34-36 |
| **IN19SI08** | **Irrigation water** | **River** | **South-west Slovenia location 2** | **2019** | **NT** | **28** |
| IN20SI01 | Irrigation water | Underground | Central Slovenia location 2 | 2020 | NT | 38 |
| **IN20SI02** | **Area** | **River** | **Central Slovenia location 2** | **2020** | **NT** | **28** |
| **IN20SI03** | **Irrigation water** | **Pond** | **South-west Slovenia location 3** | **2020** | **NT** | **28** |
| IN20SI04 | Irrigation water | Underground | South-west Slovenia location 4 | 2020 | NT | 38 |
| IN20SI05 | Irrigation water | Tap water | South-west Slovenia location 5 | 2020 | NT | 37 |
| **IN20SI07** | **Irrigation water** | **River** | **South-west Slovenia location 6** | **2020** | **NT** | **30** |
| IN20SI08 | Irrigation water | Underground | South-east Slovenia location 1 | 2020 | NT | 38 |
| **IN20SI09** | **Irrigation water** | **River** | **South-east Slovenia location 2** | **2020** | **NT** | **29** |
| **D691/21** | **Irrigation water** | **Unknown** | **North-east Slovenia location 1** | **2021** | **Undet** | **32^c^** |
| D902/22 | Irrigation water | Unknown | North-east Slovenia location 2 | 2022 | Undet | Undet |

Undet, No signal obtained with RT-qPCR; NT, not tested.

^a^The presence of ToBRFV RNA in water samples was investigated using one-step real-time quantitative reverse transcription PCR (RT-qPCR) using primers and probes from Menzel and Winter (2021), except for samples IN20SI01 and IN20SI02 where the presence of ToBRFV was tested by ISF-ISHI-Veg RT-qPCR test (ISHI-Veg 2019) and data of CaTa28 amplicon are shown.

^b^ToBRFV was detected also by high-throughput sequencing in a previous study (Bačnik et al., 2020)

^c^ToBRFV confirmed with ISF-ISHI-Veg RT-qPCR, and also with sequencing of ToBRFV specific RT-PCR product obtained with Panno et al. (2019) primers and tobamovirus generic nested PCR products obtained with primers Dovas et al. (2004).

Literature cited:

Bačnik, K., Kutnjak, D., Pecman, A., Mehle, N., Žnidarič, M. T., Aguirre, I. G., et al. (2020). Viromics and infectivity analysis reveal the release of infective plant viruses from wastewater into the environment. *Water Res.*, 115628. doi: https://doi.org/10.1016/j.watres.2020.115628.

Dovas, C. I., Efthimiou, K., and Katis, N. I. (2004). Generic detection and differentiation of tobamoviruses by a spot nested RT-PCR-RFLP using dI-containing primers along with homologous dG-containing primers. *J. Virol. Methods* 117, 137–144. doi: 10.1016/j.jviromet.2004.01.004.

ISHI-Veg (2019). Detection of Infectious Tomato brown rugose fruit virus (ToBRFV ) in Tomato and Pepper Seed -version 1. *ISF, Int. Seed Fed.* Available at: https://www.worldseed.org/wp-content/uploads/2019/04/Tomato-ToBRFV_CPP_2019.pdf.

Menzel, W., and Winter, S. (2021). Identification of novel and known tobamoviruses in tomato and other solanaceous crops using a new pair of generic primers and development of a specific RT-qPCR for ToBRFV. in *VI International Symposium on Tomato Diseases: Managing Tomato Diseases in the Face of Globalization and Climate Change 1316*, 143–148. doi: 10.17660/ActaHortic.2021.1316.20.

Panno, S., Caruso, A. G., and Davino, S. (2019). First Report of Tomato Brown Rugose Fruit Virus on Tomato Crops in Italy. *Plant Dis.* 103, 1443. doi: 10.1094/PDIS-12-18-2254-PDN.
